# Case Report: Two Novel Frameshift Mutations in *SLC20A2* and One Novel Splice Donor Mutation in *PDGFB* Associated With Primary Familial Brain Calcification

**DOI:** 10.3389/fgene.2021.643452

**Published:** 2021-05-07

**Authors:** Yuqi Shen, Shi Shu, Yaqiong Ren, Weibo Xia, Jianhua Chen, Liling Dong, Haijun Ge, Shiqi Fan, Lei Shi, Bin Peng, Xue Zhang

**Affiliations:** ^1^McKusick-Zhang Center for Genetic Medicine, State Key Laboratory of Medical Molecular Biology, Institute of Basic Medical Sciences, Chinese Academy of Medical Sciences & Peking Union Medical College (CAMS&PUMC), Beijing, China; ^2^Department of Neurology, Peking Union Medical College Hospital (PUMCH), CAMS&PUMC, Beijing, China; ^3^Department of Endocrinology, Key Laboratory of Endocrinology, Ministry of Health, PUMCH, CAMS&PUMC, Beijing, China; ^4^National Health Commission (NHC) and CAMS Key Laboratory of Molecular Probe and Targeted Theranostics, Harbin Medical University, Harbin, China

**Keywords:** primary familial brain calcification, PFBC, *SLC20A2*, *PDGFB*, mutation

## Abstract

Primary familial brain calcification (PFBC, OMIM#213600), also known as Fahr's disease, is characterized by bilateral and symmetric brain calcification in the basal ganglia (globus pallidus, caudate nucleus, and putamen), thalamus, subcortical white matter, and cerebellum. PFBC can be caused by loss-of-function mutations in any of the six known causative genes. The most common clinical manifestations include movement disorders, cognitive impairment, and neuropsychiatric signs that gradually emerge in middle-aged patients. To broaden the PFBC mutation spectrum, we examined nine members of a family with PFBC and two sporadic cases from clinical departments, and sequenced all PFBC-causative genes in the index case. Two novel frameshift mutations in *SLC20A2* [NM_001257180.2; c.806delC, p.(Pro269Glnfs^*^49) and c.1154delG, p.(Ser385Ilefs^*^70)] and one novel splice donor site mutation (NM_002608.4, c.456+1G>C, r.436_456del) in *PDGFB* were identified in the patient cohort. c.806delC co-segregated with brain calcification and led to *SLC20A2* haploinsufficiency among the affected family members. The c.456+1G>C mutation in *PDGFB* resulted in aberrant mRNA splicing, thereby forming mature transcripts containing an in-frame 21 base pair (bp) deletion, which might create a stably truncated protein [p.(Val146_Gln152del)] and exert a dominant negative effect on wild-type PDGFB. All three mutations were located in highly conserved regions among multiple species and predicted to be pathogenic, as evaluated by at least eight common genetic variation scoring systems. This study identified three novel mutations in *SLC20A2* and *PDGFB*, which broadened and enriched the PFBC mutation spectrum.

## Introduction

Primary familial brain calcification (PFBC, OMIM #213600) is a rare neurodegenerative disorder characterized by vascular calcification affecting multiple brain regions, particularly the basal ganglia, thalamus, subcortical white matter, and cerebellum (Nicolas et al., 2013a, 2015; Tadic et al., 2015; Batla et al., 2017). Brain calcification first appears in the globus pallidus, caudate nucleus, and putamen, and progressively affects the thalamus, hypothalamus, subcortical white matter, cerebral cortex, and dentate gyrus of the cerebellum (Kimura et al., 2016; Paucar et al., 2016, 2017). Clinical symptoms include cognitive impairment, psychiatric symptoms, and movement disorders (Nicolas et al., 2013a, 2015; Grangeon et al., 2019). Most patients have no obvious neurological manifestations before their middle age; however, ~60–70% of patients may exhibit progressive motor coordination dysfunction and neuropsychiatric signs, such as tremor paralysis, dystonia, ataxia, dementia, aphasia, mental confusion, and chronic headache, after the age of 40 years (Donzuso et al., 2019; Grangeon et al., 2019; Westenberger et al., 2019).

Genetically, loss-of-function mutations in *SLC20A2* cause familial and sporadic cases of PFBC (Hsu et al., 2013; Lemos et al., 2015). *SLC20A2* mutations can impair the inward transport of phosphate through loss-of-function (Wang et al., 2012), dominant negative effects (Kimura et al., 2016; Larsen et al., 2017), or haploinsufficiency mechanisms (Zhang et al., 2013). In addition, PIT-1/*SLC20A1* (Inden et al., 2016) and *XPR1* (Giovannini et al., 2013; Legati et al., 2015) are important for regulating phosphate homeostasis in the brain. PIT-2 and PIT-1 proteins belong to the type III sodium-dependent phosphate co-transporter family that mediates phosphate influx (Li et al., 2006; Crouthamel et al., 2013), whereas XPR1 is the only known transporter for phosphate efflux (Legati et al., 2015). *PDGFRB* (Nicolas et al., 2013b), *PDGFB* (Keller et al., 2013), *MYORG* (Yao et al., 2018), and *JAM2* (Cen et al., 2020; Schottlaender et al., 2020) are four known PFBC-causative genes, all of which may help maintain the structural integrity of the neurovascular unit (NVU) and regulate the permeability of the blood-brain barrier (BBB) (Westenberger et al., 2019; Zarb et al., 2019a).

To date, *SLC20A2* and *PDGFB* are most frequently involved in familial cases of autosomal dominant (AD) PFBC (Batla et al., 2017; Donzuso et al., 2019), whereas the presence of bi-allelic mutations in *MYORG* is the major cause of recessive PFBC (Bauer et al., 2019). This study aimed to identify novel pathogenic mutations in six known PFBC-causative genes and to provide new insights into the clinical diagnosis of PFBC.

## Case Description

One Chinese family and two Chinese sporadic cases of PFBC were recruited from the Peking Union Medical College Hospital (PUMCH). The eligibility criteria for patient enrollment were: (1) bilateral and symmetrical basal ganglia calcification; and (2) PFBC-related neurological symptoms. Exclusion criteria were: (1) individuals with blood biochemical disorders related to calcium, phosphate, alkaline phosphatase (ALP), or parathyroid hormone (PTH) metabolism; (2) traumatic brain injuries; (3) parasitic or viral infections; and (4) physiological and senile calcification. Written informed consent was obtained from each participant, and ethical approval was obtained from the Institutional Ethics Committee of Peking Union Medical College, Chinese Academy of Medical Sciences (CAMS&PUMC). Subsequently, the recruited patients underwent systemic physical, neurological, and blood biochemical examinations. Brain computed tomography (CT) or magnetic resonance imaging (MRI) scans were routinely adopted as part of the diagnostic workup for evidence of brain calcification and other intracranial abnormalities. All clinical examinations and diagnoses were carefully evaluated and revised by the relevant experts.

### Case-1: HB-PFBC Family

**Figure 1:**
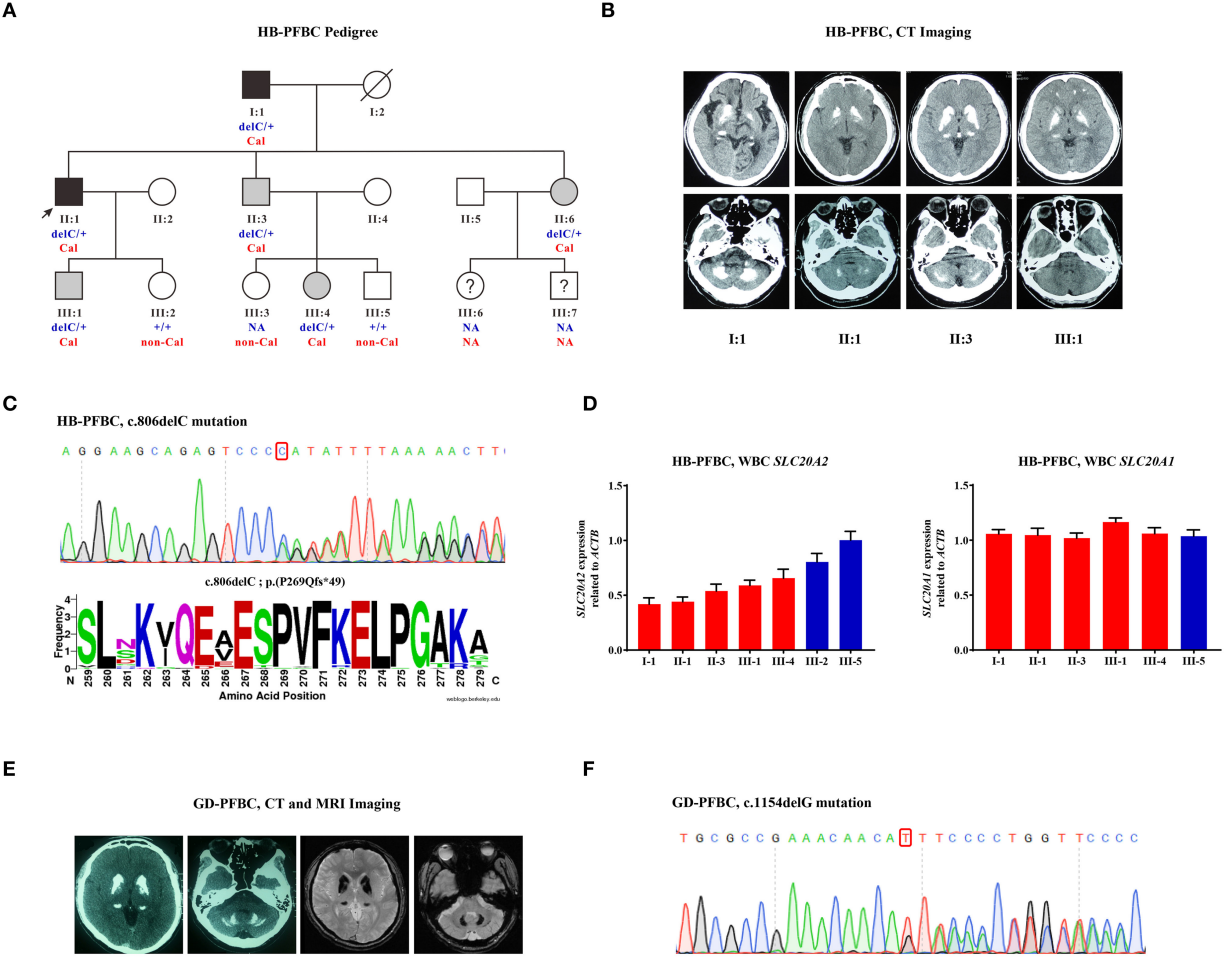
**(A)** HB-PFBC pedigree. Filled symbols represent family members affected with brain calcification, including both symptomatic (black) and asymptomatic (gray) cases. The black arrow indicates the proband and question marks indicate individuals whose blood samples and brain imaging were both not available. delC: c.806delC; +: wild-type allele; Cal, calcification; non-Cal, no calcification; NA, not available. **(B)** Brain computed tomography (CT) images of affected individuals in the HB-PFBC family. High radiopacity and density areas represent brain calcification. **(C)** The c.806delC mutation of *SLC20A2* in the HB-PFBC proband and frequency diagram of protein conservation analysis for mutated Pro269 site. **(D)** *SLC20A2* and *SLC20A1* mRNA expression in the peripheral leukocytes of the HB-PFBC family members. Red bar indicates the patient, and blue indicates normal people. **(E)** Brain CT and MRI axial T2*GRE images of the GD-PFBC patient. **(F)** The c.1154delG mutation of *SLC20A2* in the GD-PFBC patient.

A three-generation HB-PFBC family was recruited from the Department of Endocrinology, PUMCH (Figure 1A). The regions of brain calcification were revealed using intracranial CT scanning, and the high radiopacity and density areas inside the brain parenchyma represented calcification. Members (I-1, II-1, II-3, and III-1) of the HB-PFBC family, who underwent CT scanning with the imaging authorized to researchers, showed symmetric and bilateral calcification in the caudate nuclei, globus pallidus, and putamen regions, whereas no retina/lens calcification, microphthalmia, or cataracts were observed (Figure 1B). The molecular and clinical characteristics of the patients are summarized in Table 1. The old father (I-1) showed prominent and severe calcification in the cerebellar hemisphere and vermis, as well as in the hippocampus, which is seldom calcified in PFBC patients. His neurological symptoms included parkinsonism, cerebral infarction, dysarthria, gait rigidity, and ataxia. The proband II-1 reported suffering from chronic and repetitive dizziness for 2 years. Biochemical tests showed that only total thyroxine (TT4) levels were slightly lower than normal; however, PTH levels were much higher than those in the reference (Supplementary Table 1). Mutational analysis of the four known AD PFBC-causative genes revealed the c.806delC mutation in *SLC20A2* of proband II-1 (Figure 1C) that co-segregated with brain calcification in this family (Supplementary Figure 1). The c.806delC mutation is located in highly conserved regions of exon 7 of *SLC20A2* (NM_001257180.2) in multiple species (Figure 1C), theoretically resulting in a prematurely terminated mRNA transcript, which might activate a surveillance mechanism called nonsense-mediated mRNA decay (NMD) (Khajavi et al., 2006), or probably create a C-terminal truncated PIT-2 protein [p.(Pro269Glnfs^*^49)] with impaired phosphate transport function. We evaluated the impact of the c.806delC mutation on *SLC20A2* mRNA expression and found a 40–65% relative level in heterozygous carriers compared with controls, confirming that *SLC20A2* haploinsufficiency causes brain calcification in the HB-PFBC family members (Figure 1D). Furthermore, *SLC20A1*, which is considered to play an essential role in inorganic phosphate-induced cardiovascular calcification (Li et al., 2006), showed no compensation effect for the c.806delC mutation in *SLC20A2* (Figure 1D).

**Table 1:** Molecular and clinical findings in the HB-PFBC family.

**Index**	**Age^a^**	**Sex^b^**	**Mutation**	**Calcification regions**	**Symptoms**
I:1	69	M	c.806delC	Caudate nuclei, globus pallidus, putamen, thalamus, cerebral cortex, cerebellum, vermis, hippocampus	Parkinsonism, cerebral infarction, dysarthria, gait rigidity, ataxia
II:1	43	M	c.806delC	Caudate nuclei, globus pallidus, putamen, cerebellum	Chronic and repetitive dizziness
II:3	41	M	c.806delC	Caudate nuclei, globus pallidus, putamen, thalamus, cerebral cortex, cerebellum	Asymptomatic
II:6	39	F	c.806delC	Brain calcifications	Asymptomatic
III:1	19	M	c.806delC	Caudate nuclei, globus pallidus, putamen, thalamus, cerebral cortex	Asymptomatic
III:4	12	F	c.806delC	Brain calcifications	Asymptomatic
III-2	14	F	Ref. Allele	No brain calcification	Chronic headache
III-3	14	F	Not test	No brain calcification	Normal
III-5	9	M	Ref. Allele	No brain calcification	Normal

a *Age indicates age at diagnosis*;

b *M and F indicate male and female, respectively*.

### Case-2: GD-PFBC Sporadic Case

A case of a patient with sporadic PFBC, named GD-PFBC, was recruited from the Department of Neurology, PUMCH. The patient was a 45-year-old man who had been suffering from involuntary movement of his left limbs and bradykinesia for 3 years. No headache, dizziness, nausea, vomiting, convulsion, language or speech problems, or consciousness disorders were present. A family history was also referred to. Systemic physical and neurological examinations revealed no abnormalities. Serum calcium, phosphate, and PTH concentrations and thyroid function were normal. The total serum cholesterol was normal; however, triglycerides (TG: 4.78 mmol/L, normal: 0.56–1.70 mmol/L), high-density lipoproteins (HDL-C: 0.75 mmol/L, normal: 1.16–1.42 mmol/L), low-density lipoproteins (LDL-C: 2.56 mmol/L, normal: 2.70–4.10 mmol/L), and apolipoprotein A-I (apoA-I: 0.90 g/L, normal: 1.20–1.60 g/L) were all out of reference, implicating dyslipidemia. Serum homocysteine (HCY: 36.9 μmol/L, normal: 4.0–17.0 μmol/L) and blood lactic acid (2.50 mmol/L, normal: 0.50–2.20 mmol/L) levels were higher than normal values. Abdominal ultrasonography showed mild fatty lesions in the liver and prostatic calcification. Brain CT imaging revealed bilateral symmetric calcification in the caudate nuclei, lentiform nuclei, thalami, and dentate nuclei of the cerebellum (Figure 1E). Brain MRI axial T2^*^GRE also showed bilateral symmetric hypointense signal changes in the aforementioned brain regions (Figure 1E). The patient was eventually diagnosed with Fahr's disease with hypertriglyceridemia and hyperhomocysteinemia. We identified a novel c.1154delG mutation at exon 8 of *SLC20A2* (NM_001257180.2) (Figure 1F), in an evolutionarily conserved region, and predicted the formation of an NMD-directed degraded transcript or a truncated protein [p.(Ser385Ilefs^*^70)] in this patient.

### Case-3: HLJ-PFBC Sporadic Case

**Figure 2:**
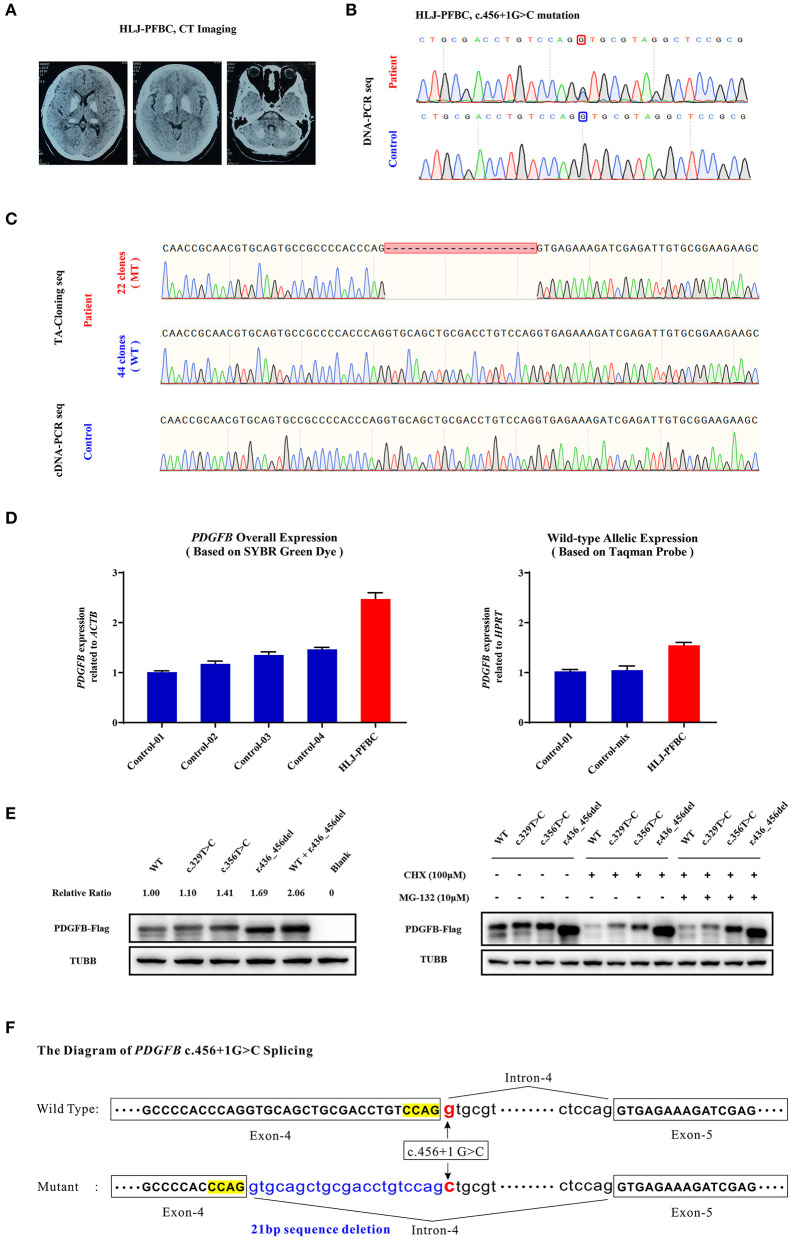
**(A)** Brain CT images of the HLJ-PFBC patient. **(B)** The *PDGFB* c.456+1G>C mutation in the HLJ-PFBC patient and normal control. **(C)** The impact of the *PDGFB* c.456+1G>C mutation on its mRNA splicing revealed by TA-cloning sequencing and cDNA-PCR sequencing in the HLJ-PFBC patient and normal control. **(D)** The overall and wild-type allele-specific expression of *PDGFB* in the peripheral leukocytes of the HLJ-PFBC patient (red) and normal controls revealed by SYBR Green dye- and TaqMan probe-based assays. Control-mix indicated a mixture of cDNA templates from six other normal controls. **(E)** Fusion protein expression of the *PDGFB*-*Flag* construct in the HEK293T cell line. The c.329T>C and c.356T>C of *PDGFB* are two previously reported PFBC-causative mutations. CHX (cycloheximide), a protein synthesis inhibitor; MG132, a ubiquitin-proteasome inhibitor. **(F)** The sketch of the mRNA splicing process of *PDGFB* c.456+1G>C.

The HLJ-PFBC patient in this case was a 46-year-old woman who complained of chronic headache, nausea, and slow writing in the 3 years prior to examination. Her physical examination revealed slightly elevated blood pressure (130/100 mmHg), and biochemical examination showed normal levels of serum calcium, phosphate, magnesium, calcitonin, and PTH. However, cranial CT revealed bilateral and symmetrical calcification in the basal ganglia, thalamus, and cerebellum (Figure 2A). A splice donor mutation (c.456+1G>C), located at the initiation of intron 4 of *PDGFB* (NM_002608.4), was suspected to be the causative mutation in this patient (Figure 2B). Mutational effects on mRNA splicing evaluated via cDNA-PCR and TA-cloning sequencing revealed that the c.456+1G>C mutation resulted in an in-frame deletion of 21 bp of nucleotides *in vivo* (Figure 2C). The relatively lower numbers of mutant TA clones (mt/wt clones = 22/44) and lower heights of cDNA sequencing peaks indicated that the newly deleted transcript was either undergoing the NMD process, the compensatory effect of the wild-type allele, or both, in the peripheral blood. Quantitative real-time PCR using SYBR Green dye and TaqMan probes revealed that the overall expression of *PDGFB* mRNA was increased by 2.47-fold when compared with age- and sex-matched controls, while the expression of the wild-type allele was increased by more than 3-fold (1.55 vs. 0.50%) (Figure 2D). Mutant PDGFB protein might be more stable than wild-type PDGFB and may induce the compensatory expression of wild-type protein in the HEK293T cell line (Figure 2E). The c.456+1G>C mutation effect on the *PDGFB* mRNA splicing process is illustrated in Figure 2F.

## Discussion

### Clinical Phenotype, Genetic Heterogeneity, and Genetic Complexity in Brain Calcification

Loss-of-function mutations in *SLC20A2* are the major genetic causes of familial and sporadic PFBC (Hsu et al., 2013; Lemos et al., 2015). Most patients are asymptomatic before the age of 40 years. Notably, among members of the HB-PFBC family, *SLC20A2* mRNA levels of III-2 (no calcification and no c.806delC mutation) were reduced to 80% of that of III-5 normal controls (Figure 1D). III-2 also presented with short and thick fingers and toes, and reported chronic headache and arthritis of the fingers. Biochemical results of proband II-1 in the HB-PFBC family showed slightly lower TT4 levels but significantly higher PTH levels than the reference range (Supplementary Table 1). Generally, TT4 decreases along with serum thyroid-binding globulin due to the intake of diazepam, testosterone, glucocorticoids, or other drugs. PTH, fibroblast growth factor-23 (FGF-23), and 1,25-dihydroxyvitamin D (1,25(OH)_2_D) are the main phosphate regulators in human physiology. PTH can increase renal phosphate excretion by reducing the expression of sodium-dependent phosphate co-transporters NaPi-IIa and NaPi-IIc in the proximal renal tubules (Bergwitz and Juppner, 2010). Elevated PTH is commonly regarded as a sub-clinical stage of hyperparathyroidism (OMIM#145000) or pseudo-hypoparathyroidism (OMIM#103580). Hypoparathyroidism, another clinical condition that can also lead to intracranial calcification, is usually caused by inadvertent removal of or accidental injury to the parathyroid gland during neck-related surgery, or by autoimmune disorders (Mannstadt et al., 2017; Gafni and Collins, 2019). Pseudo-hypoparathyroidism results from resistance to the biological effects of PTH in the peripheral organs. Both diseases share common biochemical features, such as hypocalcemia and hyperphosphatemia (Mantovani et al., 2018). Environmental factors and other genetic or epigenetic modifiers may also influence *SLC20A2* expression, leading to recurrent headaches or repetitive dizziness.

The GD-PFBC patient showed moderate to severe hypertriglyceridemia and hyperhomocysteinemia, both of which are traditional risk factors for cardiovascular diseases (CVDs). Genetic variations and other secondary acquired factors could affect serum or plasma TG, HDL-C, LDL-C, and apoA-I levels, thereby modifying the risk of CVDs (Miller et al., 2011; Nordestgaard and Tybjaerg-Hansen, 2011; Nordestgaard, 2016; Ference et al., 2017; Rosenson et al., 2018). Elevated HCY levels are generally considered to be independent and strong risk factors for the incidence and progression of coronary artery calcification (Kullo et al., 2006; Karger et al., 2020), aortic calcification (Hirose et al., 2001; Karger et al., 2020), intracranial arterial calcification, and cerebral atherosclerosis (Kim et al., 2016). Hyperhomocysteinemia can induce vascular smooth muscle cell osteogenic differentiation and calcification, and increase endothelial cell apoptosis and vascular inflammation, all of which are adverse and pathogenic cardiovascular events (Hofmann et al., 2001; Van Campenhout et al., 2009; Fang et al., 2015; Zhu et al., 2019). High plasma homocysteine could also decrease HDL-C levels by enhancing its clearance and inhibiting apoA-I protein synthesis (Liao et al., 2006), which is in accordance with the lower HDL-C levels in patients with GD-PFBC. In addition, hyperhomocysteinemia might independently result in multifocal calcifications in the brain and coronary arteries, as revealed by brain and coronary CT scans in human patients (Nah and Kim, 2010). Homocysteine metabolism is largely dependent on the folate and methionine-homocysteine cycles (McCully, 1996; Welch and Loscalzo, 1998; Hankey and Eikelboom, 1999). Genetic variations in methylenetetrahydrofolate reductase encoded by the *MTHFR* gene could lead to variations in HCY concentrations at the individual and population levels (Frosst et al., 1995; Klerk et al., 2002; Selzer et al., 2003). In the GD-PFBC case, no common hypomorphic alleles were detected in four key enzymes closely associated with folate and methionine-homocysteine metabolism (*MTHFR*, 677C>T, 1298A>C; *MTR*, 2756A>G; *MTRR*, 66A>G; *CBS*, 844 ins68) (Kluijtmans et al., 2003; Moll and Varga, 2015). Treatment of this patient with vitamin B supplements (vitamin B6, B12, and folic acid) might alleviate hyperhomocysteinemia and improve clinical outcomes. However, dyslipidemia and hypertriglyceridemia, which are closely associated with critical cardiovascular events, such as mitral annular and aortic valve calcification and coronary artery calcification (Greif et al., 2013; Thanassoulis et al., 2013; Afshar et al., 2017; Zheng et al., 2019), also deserve special attention in this patient. We could not perform an informative analysis in the GD-PFBC patient due to the non-availability of RNA samples. However, we evaluated the pathogenicity of the c.1154delG variation in *SLC20A2* (NM_001257180.2) in ten function-predicting scoring systems (ACMG, DDIG-in, FATHMM-Indel, MutPred-LOF, PROVEAN, SIFT-Indel, CADD/CADD-Splice, CAPICE/GAVIN, MutationTaster, and VEST/VEST-Indel), three software programs providing conservation scores (GERP++, phyloP100way, and phastCons100way), and four variation databases (gnomAD, ExAC, dbSNP, and 1000 Genomes Project), most of which supported the deleterious effect of this variation (Table 2).

**Table 2:** Computational evidence for the pathogenicity of three novel variations.

**Variation/Gene^a^**	**c.806delC, *SLC20A2***	**c.1154delG, *SLC20A2***	**c.456+1G>C, *PDGFB***
**Position (Hg38)**	Chr8: 42439578	Chr8: 42437358	Chr22: 39231621
**Transcript ID**	NM_001257180.2	NM_001257180.2	NM_002608.4
**Exon**	Exon 7 of 11	Exon 8 of 11	Intron 4 of 6
**Protein^a^**	p.(Pro269Glnfs*49)	p.(Ser385Ilefs*70)	p.(Val146_Gln152del)
**ACMG**	5, Pathogenic	5, Pathogenic	5, Pathogenic
DDIG-in	0.924259, Disease	0.936076, Disease	—
FATHMM-Indel^b^	0.9765, Pathogenic	0.9945, Pathogenic	—
MutPred-LOF	0.58397, Pathogenic	0.57958, Pathogenic	—
PROVEAN^b^	NA^d^	NA*^d^*	—
SIFT-Indel	Damaging	Damaging	—
FATHMM-MKL	—	—	0.9714, Deleterious
BayesDel^b^	Not tested	Not tested	0.5693, Pathogenic
CADD/CADD-Splice^b^	32, Deleterious	32, Deleterious	34, Deleterious
CAPICE/GAVIN^b^	0.7224, Pathogenic	0.2847, Pathogenic	0.2293, Pathogenic
DANN^b^	Not tested	Not tested	0.9908, Deleterious
Eigen	Not tested	Not tested	13.21783, Pathogenic
MutationTaster	Disease causing	Disease causing	Disease causing
VEST/VEST-Indel	0.917, Pathogenic	0.955, Pathogenic	0.927, Pathogenic
GERP++^b^	6.060, Conserved	5.630, Conserved	5.270, Conserved
phyloP100way	9.012, Conserved	7.726, Conserved	7.279, Conserved
phastCons100way	1.000, Conserved	1.000, Conserved	1.000, Conserved
gnomAD^c^	Absent	Absent	Absent
ExAC^c^	Absent	Absent	Absent
dbSNP^c^	Absent	Absent	Absent
1KGP^c^	Absent	Absent	Absent

a *Variations named according to current recommendations of the Human Genome Variant Society (http://www.HGVS.org/varnomen)*.

b *Pathogenicity Threshold: FATHMM-Indel > 0.967; PROVEAN < −2.5; BayesDel > 0.0692655; CADD/CADD-Splice > 20; CAPICE/GAVIN > 0.02; DANN > 0.97; Cut-offs for other prediction tools > 0.5 or no score provided. Conservation threshold: GERP++ > 2*.

c *gnomAD, The Genome Aggregation Database; ExAC, The Exome Aggregation Consortium; dbSNP, The Single Nucleotide Polymorphism Database; 1KGP, The 1,000 Genomes Project*.

d *NA, Not available*.

The HLJ-PFBC patient, harboring the c.456+1G>C mutation of *PDGFB*, presented with nausea and higher blood pressure, which has rarely been reported previously. It seemed that her nausea was the result of elevated blood pressure because no other risk factors were found for this symptom. The *PDGFB* gene encodes platelet-derived growth factor beta, which is functionally activated when forming PDGF-BB homodimers or PDGF-AB heterodimers with PDGFA (Betsholtz and Keller, 2014). Loss-of-function mutations in *PDGFB* could affect its synthesis, maturation, and dimerization, resulting in impairment of the PDGFB-PDGFRB pathway and dysfunction of the BBB, eventually leading to PFBC (Vanlandewijck et al., 2015). We confirmed that the c.456+1G>C mutation removed the canonical 5-prime splice donor site and resulted in aberrant mRNA splicing, creating an in-frame deleted transcript. A similar splice donor site mutation, c.456+1G>A, was reported in a nuclear family, and predicted to lead to exon 4 (NM_002608.4) skipping and introduction of a frameshift version of PDGFB. However, both patients in the nuclear family had a severe migraine, a history of depression, and calcification in the basal ganglia, thalamus (only mother affected), cerebral cortex (only proband affected), and subcortical white matter (Ramos et al., 2018). Another splice acceptor site mutation in the same intron (c.457-1G>T) may lead to exon 5 (NM_002608.4) skipping and frameshift protein in carriers, leading to chronic headache and intellectual disability (Sekine et al., 2019). These results suggest that different substitutions on the same splicing unit (GT-AG in DNA code) could result in distinct molecular events and disease phenotypes. Of note, another three splice site mutations in *PDGFB* (c.64-3C>G, c.160+2T>A, and c.602-1G>T) also resulted in aberrant splicing processes and frameshift outcomes (Nicolas et al., 2015; Koyama et al., 2017; Sekine et al., 2019). In our study, the c.456+1G>C variant was functionally deleterious, as revealed by the comprehensive bioinformatic analyses (Table 2), and could activate the upstream cryptic splice donor site residing in exon 4 and create a novel mature transcript with an in-frame 21 bp deletion (NM_002608.4, r.436_456del). This novel transcript and its translated protein might be more stable than and promote the compensatory expression of the wild-type mRNA and protein in the peripheral blood and HEK293T cell line. The mutant PDGFB protein was resistant to degradation, perhaps via the lysosomal pathway, but not the ubiquitin-proteasome system, as revealed by the combinational post-treatment HEK293T cell line with cycloheximide and MG132 (Ostman et al., 1992). These results suggest that the mutant PDGFB protein might impair the PDGFB-PDGFRB pathway by disrupting disulfide bond formation and affecting its dimerization with wild-type PDGFB protein, thus producing a dominant negative effect (Shim et al., 2010). However, the exact and real molecular and functional effects of the c.456+1G>C mutation in the brain of patients with PFBC remain unclear.

Patients with PFBC can exhibit peripheral calcification, presenting as skin microangiopathy (Biancheri et al., 2016; Nicolas et al., 2017). Intracranial senile calcification is a common neuroimaging sign in healthy individuals. During the human lifespan, the choroid plexus, pineal gland, and habenular nuclei tend to accumulate physiologic calcium and phosphate, which is likely due to organ functional decline or insufficient hormone concentration (Grech et al., 2012). In addition, brain calcification load and location show significant inter-individual differences in PFBC patients and mouse models (Zarb et al., 2019b).

### *SLC20A2* and *PDGFB* Dosage and Functional Effect in Brain Calcification

*SLC20A2* gene haploinsufficiency is a likely pathogenic mechanism of brain calcification; half dosage of *SLC20A2* expression cannot maintain the phosphate transport demand in the brain (Baker et al., 2014; Fujioka et al., 2015; Guo et al., 2019; Mu et al., 2019). Deletions adjacent to the regulatory element in the *SLC20A2* coding region may also cause PFBC (Pasanen et al., 2017; Cassinari et al., 2020). *SLC20A2* expression might be related to the severity of brain calcification to some extent, such as the genetic dosage effect observed in patients harboring *MYORG* mutations (Chen et al., 2019b, 2020; Grangeon et al., 2019). Some studies have also suggested a dominant negative function of *SLC20A2* genetic variations (Kimura et al., 2016; Larsen et al., 2017), with bi-allelic pathogenic mutations in *SLC20A2*, resulting in more severe brain calcification (de Oliveira et al., 2013; Chen et al., 2019a). Phosphoric acid transport activities were significantly maintained in the presence of the c.680C>T mutation in *SLC20A2*. However, those harboring this mutation revealed severe and bilateral basal ganglia calcification (Nishii et al., 2019), suggesting that this mutation might exert a dominant negative effect, as seen in *SLC20A2* variants encoding D28N, H502A, and E575K (Larsen et al., 2017). The effect of *SLC20A2* dosage on PFBC remains controversial, and environmental factors and genetic or epigenetic modifiers need to be taken into consideration. PFBC-causative mutations in *PDGFB* might lead to complete loss of *PDGFB* function either through abolished protein synthesis or defective stimulation of PDGFRB and its downstream pathways (Vanlandewijck et al., 2015). However, some discrepancies were observed, such as when *Pdgfrb*^redeye/redeye^ mice, which showed nearly complete reduction of PDGFB-PDGFRB signaling, did not develop brain calcification (Vanlandewijck et al., 2015). The relationship between impaired PDGFB-PDGFRB signaling and brain calcification requires further examination.

## Conclusion

As of the end of January 2021, according to the Human Gene Mutation Database (HGMD, www.hgmd.cf.ac.uk/ac/index.php), 142 mutations in *SLC20A2* have been identified, including 75 missense and non-sense mutations, 15 splice-site mutations, 32 small deletions, 5 small insertions and duplications, and 15 gross mutations; 24 mutations were identified in *PDGFB*, including 19 missense and non-sense mutations, three splice-site mutations, and two gross deletions. We summarized and compiled the other four pathogenic mutations in *PDGFB* from the literature (Sekine et al., 2019). In the present study, we identified two novel frameshift mutations in *SLC20A2* (c.806delC and c.1154delG) and one splice donor site mutation in *PDGFB* (c.456+1G>C), which have broadened and enriched the *SLC20A2* and *PDGFB* mutation spectrum (Supplementary Figure 2).

## Data Availability Statement

All datasets generated for this study are included in the article/Supplementary Material, further inquiries can be directed to the corresponding authors.

## Ethics Statement

The studies involving human participants were reviewed and approved by the Institutional Ethics Committee of Peking Union Medical College, Chinese Academy of Medical Sciences (CAMS& PUMC). Written informed consent to participate in this study was provided by the participants' legal guardian/next of kin.

## Author Contributions

LS, BP, and XZ conceived and designed the study. All experiments and statistical analyses were conducted by LS, YR, YS, and HG. Three novel mutations were identified by LS and YS. Clinical samples and professional medical guidance were provided by SS, WX, JC, and LD. LS, SF, and YR cooperatively prepared the original manuscript. LS, YR, SF, and XZ independently checked and revised the grammar, syntax, and logical errors. All authors listed have made a substantial, direct and intellectual contribution to the work, and approved it for publication.

## Conflict of Interest

The authors declare that the research was conducted in the absence of any commercial or financial relationships that could be construed as a potential conflict of interest.
