# Supplementary material for: Case Report: Two Novel Frameshift Mutations in SLC20A2 and One Novel Splice Donor Mutation in PDGFB Associated With Primary Familial Brain Calcification
Source: Front Genet. 2021 May 7;12:643452. doi: 10.3389/fgene.2021.643452 (PMC8138311; doi:10.3389/fgene.2021.643452)
Supplement: Supplementary file 1 [file Data_Sheet_1.docx]

Supplementary Material

# Supplementary Figures and Table

## Supplementary Figures

**Supplementary Figure S1 | c.806delC mutation in HB-PFBC family**


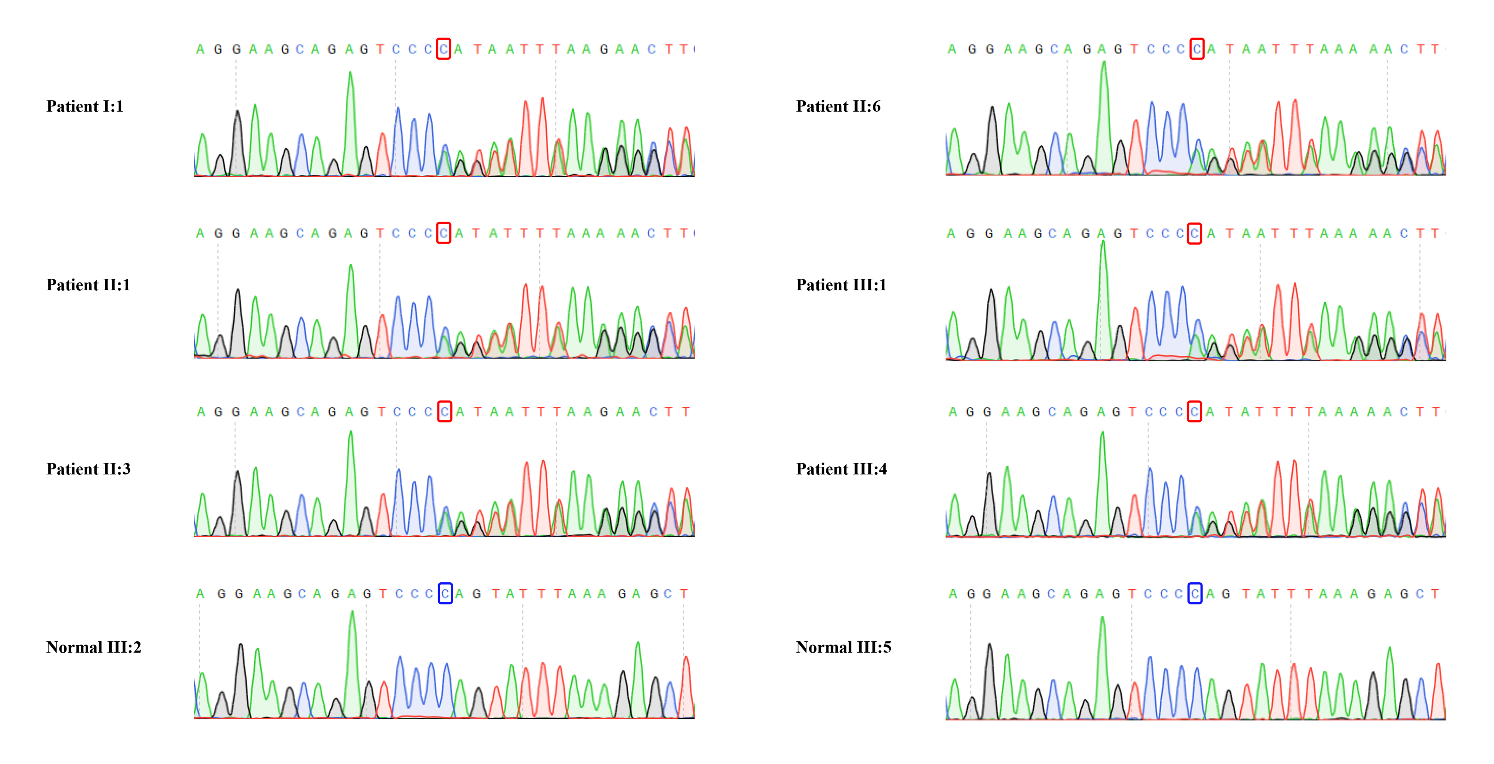


**Supplementary Figure S2 | Mutation spectrum of *SLC20A2* and *PDGFB* in PFBC**


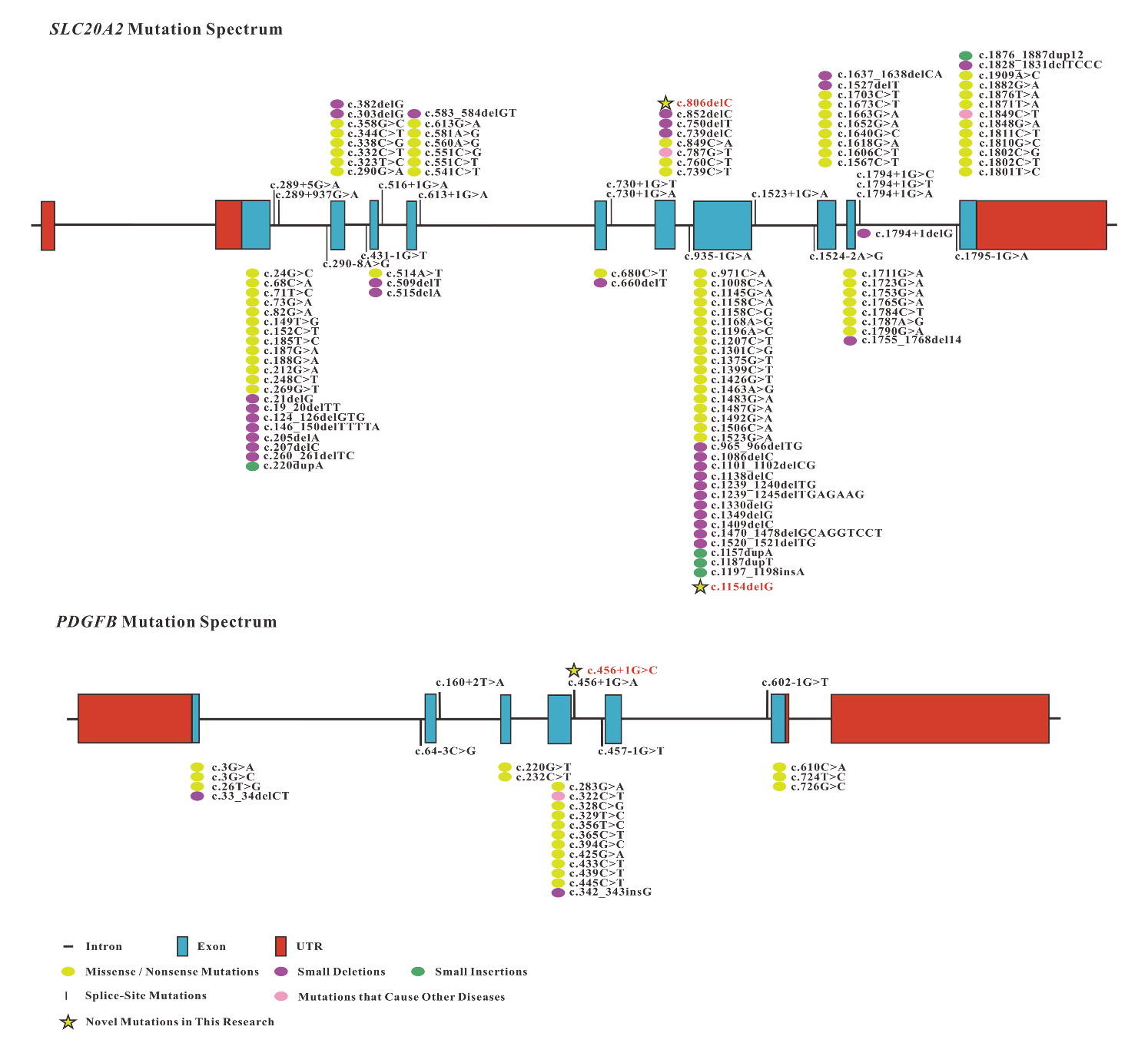


## 2．Supplementary Table

**Table S1 | Blood biochemical indexes of proband from HB-PFBC family**

| **Item** | **Results** | **Unit** | **Reference** |  | **Item** | **Results** | **Unit** | **Reference** |
| --- | --- | --- | --- | --- | --- | --- | --- | --- |
| TT3 | 1.47 | nmol/L | 0.89 ~ 2.44 |  | K | 4.0 | mmol/L | 3.5 ~ 5.5 |
| **TT4** | **56.27** | nmol/L | 62.68 ~ 150.84 |  | Na | 143 | mmol/L | 135 ~ 145 |
| TSH | 0.898 | IU/ml | 0.350 ~ 4.940 |  | Cl | 106 | mmol/L | 96 ~ 111 |
| FT3 | 4.96 | pmol/L | 2.63 ~ 5.70 |  | Ca | 2.36 | mmol/L | 2.13 ~ 2.70 |
| FT4 | 11.72 | pmol/L | 9.00 ~ 19.04 |  | Mg | 0.95 | mmol/L | 0.70 ~ 1.10 |
| TPO-Ab | 0.08 | IU/ml | 0.00 ~ 12.00 |  | P | 1.14 | mmol/L | 0.81 ~ 1.45 |
| TG-Ab | 13.98 | IU/ml | 0.00 ~ 34.00 |  | Cr (E) | 76 | umol/L | 59 ~ 104 |
| ALP | 61 | U/L | 30 ~ 120 |  | UA | 285 | umol/L | 210 ~ 416 |
| **PTH** | **110.0** | pg/ml | 12.0 ~ 65.0 |  |  |  |  |  |
